# Calcium Isotopic Composition in Reindeer ( *Rangifer tarandus* ) Bones and Tooth Enamel: A Scalable Tool to Quantify Nursing Frequency in Past and Modern Populations?

**DOI:** 10.1002/rcm.70178

**Published:** 2026-10-03

**Authors:** Auguste Hassler, Eve E. Lindroos, Lu Yang, Zoltan Mester, Kate Britton, Clément P. Bataille

**Affiliations:** ^1^ Department of Earth and Environmental Sciences, Advanced Research Complex Building University of Ottawa Ottawa Ontario Canada; ^2^ Department of Archaeology University of Aberdeen Scotland UK; ^3^ Metrology Research Centre National Research Council Canada Ottawa Ontario Canada; ^4^ Géosciences Environnement Toulouse, Observatoire Midi Pyrénées, CNRS/UMR 5563 Toulouse France; ^5^ Department of Forestry and Natural Resources Purdue University West Lafayette Indiana USA

**Keywords:** caribou, diet, pathology, sampling strategy, weaning

## Abstract

**Rationale:**

Populations of North American caribou and Eurasian reindeer (*Rangifer tarandus sspp*.), essential for Arctic ecosystems and human communities, are experiencing unprecedented declines due to climate change and anthropogenic pressures. While understanding their historical resilience and demographic dynamics is critical for conservation, long‐term population trends and the mechanisms driving these declines remain partly unresolved.

**Methods:**

This study explores if calcium (Ca) isotopic compositions (δ^44/42^Ca) measured by MC‐ICP‐MS could help quantify the number of nursing periods experienced by female reindeer during their lifetimes, as proxy for monitoring fecundity throughout the Quaternary. This pilot study focuses on five reindeer specimens who lived in controlled environmental conditions with documented life histories (including periods spent nursing). Skeletal elements with specific mineralization timings (molars and jawbones) were targeted to help untangle the effect of nursing from confounding factors, such as specimen specific diet and physiological baselines.

**Results:**

Despite this sampling approach, no clear effect of nursing was observable in jawbones δ^44/42^Ca values. We hypothesize that this may be the consequence of differences in experienced nursing activity and isotopic fractionation compared to other cases from the literature or of a faster than expected turnover rate of Ca in *Rangifer* jawbones. In parallel, insights of age‐related Ca imbalance from an undiagnosed pathology (e.g., osteoporosis) were found in one specimen, while generated data also provide complementary perspectives for investigating seasonal diet and weaning habits in *Rangifer*.

**Conclusions:**

While exploring past fecundity might prove difficult using this approach, these results are promising regarding the study of paleo‐diet and pathology. A path for future methodological improvements to address the shortcomings of our approach is outlined. This work notably underscores the urgent need for better quantifying elemental turnover rates in bones and for more controlled environment studies, two critical steps to develop robust geochemical proxies for wildlife conservation and paleoecology.

## 1 Introduction

Emerging in the early Pleistocene (earliest fossil evidence: ~1.6 Ma [1]), the history of the *Rangifer* genus (reindeer and caribou) has been profoundly influenced by the glacial cycles of the Quaternary period, with the expansions and recessions of continental icesheets shaping the migrations and demography of the group [2]. Today, *Rangifer* populations are ecological cornerstones of Arctic ecosystems in North America and northern Eurasia, representing the most abundant large mammal in these regions [3]. Beyond their ecological role, these animals hold deep cultural, economic, and subsistence significance for many northern Indigenous communities of Eurasia and North America [4, 5]. Over the past four decades, however, dramatic local population declines (e.g., the 99% collapse of the George River herd between 1993 and 2018) have contributed to a global downward trend [6, 7]. These shifts in *Rangifer* demographics and migration patterns can trigger ecosystemic ripple effects, impacting flora, apex predators, and human communities [8, 9, 10, 11]. The interplay between *Rangifer* population dynamics, human activities, and climate change is spatially heterogeneous and complex [8, 12, 13, 14, 15]. Clarifying these relationships is fundamental for developing informed conservation strategies, which in turn are critical for preserving biodiversity and the survival of northern communities and their culture.

While centuries of field observation data from local populations and biologists have advanced our understanding of *Rangifer* ecology and Arctic ecosystem dynamics, longer term processes remain less well understood in comparison. In the last decades, progress has been made in understanding long‐term *Rangifer* ecology from skeletal remains of the Quaternary prehistorical period, thanks to dental microwear, traditional and radiogenic isotopic systems (i.e., H, C, N, O, S, ^87^Sr/^86^Sr) used as proxies of diet and mobility [16, 17, 18, 19, 20, 21, 22, 23, 24]. However, past demography and population dynamics cannot be quantified using these methods, leaving uncertainties regarding the fluctuations and resilience of *Rangifer* populations beyond the time frame of historical observations. Reconstructing *Rangifer* population dynamics over the past 50 000 years (spanning the Late Pleistocene to the early Holocene) could provide critical insights into the long‐term drivers of the population changes of this genus. This period is particularly compelling, as *Rangifer* successfully adapted to massive environmental shifts, from the Last Glacial Maximum (~20 kya) to the rapid warming and increasing anthropogenic pressure that commenced with the start of the Holocene (~11 kya), a transition marked by the extinction of many other large species of the Pleistocene megafauna, such as saber‐toothed cats, giant sloths, woolly mammoths, and rhinos [25, 26, 27, 28].

Ancient DNA (aDNA) analyses of mitochondrial and nuclear microsatellite DNA have yielded estimates of effective population sizes for Pleistocene megafauna species, including *Rangifer* [28, 29, 30, 31, 32]. The value of these estimates cannot be understated, as they provide valuable insights into past and future population dynamics, particularly when combined with ecological and paleoenvironmental models. However, they face challenges such as incomplete population representativity (e.g., mtDNA reflects only female genetic history), temporal resolution constraints [29, 31], and DNA preservation biases in certain depositional environments [33]. To address these limitations, DNA‐independent methods (particularly those based on chemically resilient proxies) could further enhance demographic models by providing valuable control data while refining model priors and inputs.

One promising approach is nontraditional isotope geochemistry, which has provided insights into a growing number of (paleo‐)biological questions within past decades [34, 35, 36, 37]. Calcium (Ca) isotopic compositions in bones and teeth, for example, offer ecological (e.g., diet and trophic level), physiological (e.g., lactation and pathologies), and behavioral insights, while demonstrating remarkable resilience to post‐mortem chemical alteration [36, 38, 39, 40, 41]. Critically, Ca isotopes are linked to lactation, as the preferential incorporation of light Ca isotopes into milk enriches nursing females in heavier Ca isotopes, resulting in relatively elevated blood and/or bone ^44^Ca/^42^Ca ratios [42, 43, 44, 45, 46, 47, 48]. This pattern has been observed in sheep, mice, and cervids [43, 44, 47], with models suggesting that the isotopic shift in bones could notably correlate with the duration and number of nursing periods [45]. Thus, Ca isotopes could potentially be used to quantify the number of nursing events experienced by *Rangifer* females, offering a novel proxy for this key parameter of population dynamics.

To test this hypothesis, we designed a pilot study using *Rangifer* specimens from the controlled environment of the Large Animal Research Station (LARS) in Fairbanks (Alaska, USA), where specimens were monitored throughout their lives, including nursing periods. Previous studies have shown that the amplitude of nursing‐driven ^44^Ca enrichment in bones is moderate at the scale of a whole population, with significant overlaps between females with nursing histories and controls (males and non‐nursing females), thought to reflect dietary and physiological variability between specimens [43, 44, 47]. Direct male–female comparisons are therefore unlikely to yield quantitative relationships between Ca isotopes and nursing frequency. To overcome this, we developed a tooth enamel sampling strategy from early forming teeth to establish prenursing adult baselines for each specimen, enabling specimen‐specific comparisons with bone Ca isotopic compositions. We also optimized the sampling strategy for scalability and cost‐efficiency, limiting intratooth sampling to single points per first and third molars. Finally, we explored additional sources of isotopic variability, including juvenile milk consumption records in tooth enamel and age‐related pathologies.

## 2 Materials and Methods

### 2.1 Reindeer Sampling

In 2022, following their death by natural causes or euthanasia by decision of a veterinarian, the lower jaws of five adult reindeer specimens (2 females, 3 males), born and raised within the Large Animal Research Station (LARS) of the University of Alaska Fairbanks (UAF, USA), were opportunistically collected at the time of necropsy. The LARS is an Animal Welfare Act registered research facility (96‐R‐0001) and has an Assurance of Compliance with Public Health Service (PHS) Policy (D16‐00482). The LARS herd that studied specimens originate from is inspected by the US Department of Agriculture (USDA) and the UAF Institutional Animal Care and Use Committee (IACUC), guaranteeing high standards of ethics and animal welfare. These jaws were temporarily preserved in a freezer, then boiled (about one hour in demineralized water), mechanically cleaned from soft tissues, soaked in pure ethanol for 60 to 120 min, then left to dry under a fume hood. Relevant life details of these specimens are summarized in Table 1. These animals fed ad libidum on a mix of pelleted feed and mineral supplements during winter, with access to pasture grasses during summer. Those diet items were not analyzed for the purpose of this study, and their relative proportion in each specimen's diet is unknown. No diet supplementation was specifically provided to females, neither during gestation nor nursing periods.

**TABLE 1:** Life details and M1 crown heights of studied reindeer.

ID	Sex	DOB	DOD	Birth event dates	Reported health issue prior death	M1 crown height (cm)
E22‐083A RD 273	Female	27/04/2012	10/05/2022	(22/04/2015) 20/04/2016 08/04/2019	None, old age	0.67
E22‐083B RD 580	Female	16/04/2015	10/05/2022	20/04/2018 07/04/2019 25/04/2020	None	0.71
E22‐131 RD 608	Male	19/04/2016	29/08/2022	n/a	Recurring lameness	0.54
N22‐133 RD 853	Male	13/04/2018	12/09/2022	n/a	Died unexpectedly after being in harem	0.79
N22‐144 RD 730	Male	24/04/2017	25/09/2022	n/a	Died unexpectedly after being in harem	0.51

*Note:* Dates are in dd/mm/yyyy format. DOB and DOD respectively stand for date of birth and death. The birth event in brackets corresponds to a neonatal death resulting in no nursing during the months following the event. All other birth events involved one fawn, subsequently nursed for approximately 3‐4 months (from their birth in mid/late April to weaning in mid/late July).

Left hemimandibles were sampled for tooth and bone tissues, on the buccal side. Tooth enamel bioapatite crystallites (carbonated hydroxyapatite) grow within the span of weeks to months from their apposition to the end of their maturation, then stay largely chemically inert [49, 50, 51, 52, 53]. To represent the juvenile stage of these specimens, about 0.5 mg of enamel from the tip of the first molar cusp (M1) was sampled using a hand drill (tungsten carbide bit, Dremel). When tooth wear is limited enough, this sampling should provide enamel having mineralized in large part during the nursing prior to weaning [44, 54, 55, 56].

About 0.5 mg of enamel was similarly sampled on the third molar (M3) at 0.4 mm from the cementoenamel junction. Based on published reindeer δ^18^O records where winter and summer seasons can be distinguished [54, 57], tooth eruption patterns [56, 58, 59], radiographs [55], crown heights [60], and complemented by mule deer and red deer mineralization‐eruption lag for a rough general cervid assessment [54, 61, 62], we estimate that targeted enamel areas on the M3 would have mineralized at approximately 1–1.5 years old (assuming limited variability in enamel growth timing between specimens). Considering a regular *Rangifer* weaning age of about 6 months maximum [63, 64, 65], and 3 to 4 months for studied specimens, this sampling strategy aims to collect enamel representing a postweaning and prebreeding stage (i.e., past weaning age and before lactation and nursing would begin in sexually mature females).

Contrary to tooth enamel bioapatite crystals, most bones undergo continuous formation and resorption throughout life [66, 67]. The rate of this remodeling and associated elemental turnover changes between species, individuals, bones, bone parts, and across the lifetime of an individual [68, 69, 70, 71, 72, 73], but cortical bones will generally encompass years to decades of continuously renewed bioapatite crystals in mammals [70, 73, 74, 75, 76, 77]. *Rangifer* jawbones should therefore have aggregated bioapatite crystals from which a portion would have formed during nursing periods in studied female specimens. Therefore, about 0.5 mg of bone was sampled 1 cm below the M3 for both male and female specimens (arbitrarily selected for cross‐specimen consistency), with males constituting lactation‐free controls.

This triple sampling strategy was designed to be realistically scalable at the scale of populations in future studies. Conceptually, M1 versus M3 comparisons would contribute to support the postweaning nature of the M3 record with the aim of constituting a valid postweaning and pre‐reproduction adult baseline. Moreover, the difference between jawbone values and these M3 baselines should be a function of the number and duration of nursing periods occurring between the mineralization of the M3 and the end of bone mineralization at death, modulated by the time separating these nursing periods from the death the animal. This approach is built upon the observation that, in some conditions (notably relatively long nursing periods and sizeable milk production in regard to body mass [45]), the preferential trapping of light Ca isotopes in the milk of lactating female mammals and resulting enrichment in heavy Ca isotopes of the blood can be recorded in bones [42, 43, 45, 47, 48], notably in cervids [44].

### 2.2 Sample Preparation

Ultrapure water (resistivity ≥ 18.2 MΩ.cm at 25°C, milli‐Q, Millipore system) was used for cleaning and dilutions. A combination of ACS grade hydrochloric acid (HCl) and nitric acid (HNO_3_) was used for labware initial cleaning steps, completed by trace metal grade (TMG) HCl and HNO_3_ (or ACS grade distilled with a Savilex DST‐1000 Acid Purification System, achieving similar blank levels) for final labware cleaning steps and all steps involving direct contact with studied materials. Sample and standard preparation steps were carried out in a dust‐free environment, within the clean lab facilities of the University of Ottawa. The list of labware and cleaning procedures used in this study has been previously detailed [78].

Prior to undergoing chromatography, reindeer enamel samples were digested in 2 mL of 15 M HNO_3_ on a hot plate at 130°C for a minimum of 2 h. Reindeer bone samples were mixed with 2 mL of 15 M HNO_3_ and underwent a microwave assisted digestion (20 min heating and 30 min holding at 250°C, starting pressure 40 bar, Multiwave 7000, Anton Paar). Resulting digests were evaporated to dryness and dissolved in a few drops of 10 M HCl, before being evaporated to dryness again and conditioned for chromatography.

Reindeer samples (tooth enamel and bones) were processed by strictly following the guidelines and procedures of Hassler et al. (2026) [78] (refer to chromatography sequence A), consisting in a core matrix elution using 1 mL of AG 50W‐X12, 200‐400 mesh resin (Bio‐Rad), followed by a Sr/Ca separation with 0.3 mL of Sr resin (50‐ to 100‐μm particle size, Eichrom). Solutions collected after the Sr/Ca separation step were evaporated to dryness, and a 1‐h digestion with 0.5 mL of 15 m HNO_3_ (TMG) on hotplate at 120°C was performed to eliminate potential residual esters from the Sr resin. Four reference materials were processed and analyzed alongside these samples following the guidelines of Hassler et al. (2026) [78] and used as secondary standards to monitor chromatography and instrumental performances. This includes SRM1486 and SRM1400 (US National Institute of Standards and Technology—NIST) bioapatite standards, IAPSO seawater (Ocean Scientific International Ltd.—OSIL), and CACB‐1 calcium carbonate (National Research Council of Canada—NRC) [79]. A purified solution of calcium plasma standard solution (10 000 μg/mL, Specpure, Product no: 014407, lot no: 1359261, Thermo Scientific), referred as “alpha‐Ottawa” throughout this study and adjacent works [78], was used as an in‐house bracketing standard during Ca isotopic measurements. All reference material data presented in this study are a subset of a larger data set previously reported in Hassler et al. (2026) [78].

### 2.3 MC‐ICP‐MS Measurement

In this study, Ca isotopic compositions are reported as δ^44/42^Ca and δ^43/42^Ca values, defined as follows: δ^X/Y^Ca (‰) = (*R* ^X/Y^Ca_sample_/*R* ^X/Y^Ca_standard_) – 1) × 1000, where X and Y are two Ca isotopes and *R* is the isotope ratio of either a sample or primary standard of reference. In this study, alpha‐Ottawa is used as the primary standard for reported δCa values unless otherwise specified. The SRM915a (NIST) commonly used in the literature to express δCa values is no longer commercially available. By comparison with SRM1486 and IAPSO values from joint works and the literature (see Hassler et al., 2026 [78] and reference therein), we estimate that δ^44/42^Ca (SRM915a) = δ^44/42^Ca (alpha‐Ottawa) + 0.500 (in ‰). Using this formula, δ^44/42^Ca (SRM915a) values are also given for reference in relevant figures and tables.

Measurements of Ca isotopic compositions were carried on the Neptune plus (Thermo‐Fisher) MC‐ICP‐MS instrument hosted at the Metrology Research Centre of the NRC (Ottawa). Samples were introduced in a 2% v/v HNO_3_ (TMG) medium. Analyses were performed using the default instrumental configuration described in Hassler et al. (2026) [78], consisting of a high signal amplification and low transmission wet plasma configuration, in medium resolution mode, with Faraday cups paired with both 10^11^Ω amplifiers (for ^44^Ca^+^ and ^42^Ca^+^ beams) and 10^13^Ω amplifiers (for ^43^Ca^+^ and ^87^Sr^2+^ beams). The monitoring of the ^87^Sr^2+^ signal was used to correct Sr^2+^ interferences using the formulas described in Hassler et al. (2026) [78] and previous works [80]. δCa values reported thereafter are exclusively Sr^2+^ corrected values, although Sr^2+^ interferences were negligible thanks to the thorough Sr/Ca separation step using 50 to 100 μm particle size Sr resin [78]. Acquisition parameters, calibration methods, and corresponding limits of matrix and Sr/Ca thresholds have been previously detailed in Hassler et al. (2026) [78].

### 2.4 AI Statement

During the preparation of this manuscript, the authors used Le Chat (Mistral AI) exclusively to rephrase and condense sections of the original text written by the authors. After this tool was used, all authors reviewed and edited the manuscript and take full responsibility for the content of the publication.

## 3 Results

### 3.1 Precision and Accuracy of Ca Isotope Ratios

Procedural blanks were negligible and were consistently below 25 ng of Ca (i.e., 2000 times less than samples and standards with low Ca abundance). Average δ^44/42^Ca and δ^43/42^Ca values and respective standard deviation (SD) for the reindeer samples (Table S1) and secondary standards (Table S2) are consistent with the literature (e.g., see reviews in Delette et al., 2025 [81] and Hassler et al., 2026 [78]). The linear mass dependency relationship between δ^44/42^Ca and δ^43/42^Ca values for all samples and standards yield a slope close to the theoretical slope of 0.5067 predicted by the exponential mass‐dependent fractionation law, supporting the absence of significant interferences or uncorrected instrumental bias (Figure 1).

**FIGURE 1:**
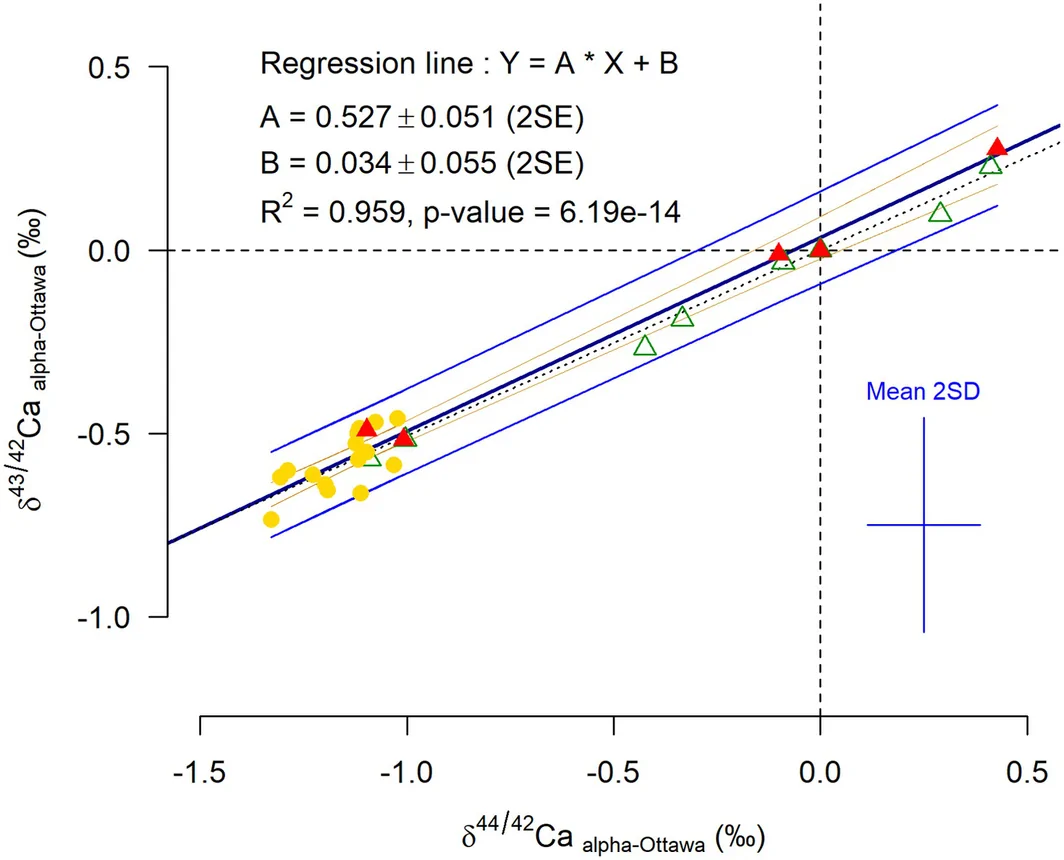
Three‐isotope plot of studied reference materials (red triangles, green open triangles) and reindeer samples (yellow circles). δCa values are expressed relative to alpha‐Ottawa bracketing standard. Open green triangles represent long‐term reference material values reported in Hassler et al. (2026) [78] and provided for reference only (excluded from the linear regression). Red triangles are a subset of these long‐term data, prepared and analyzed alongside the reindeer samples of this study. These measurements (red triangles and yellow circles only) plot on a linear regression line (dark blue, central solid line) with a slope of 0.527 ± 0.051 (2SE) with an intercept of 0.034 ± 0.055 (2SE), indistinguishable within uncertainties from the 0.5067 slope predicted from the exponential mass‐dependent fractionation law (black dotted line). The two outer blue lines delimit the prediction interval of the regression line, while the inner orange lines show its 95% confidence interval. This supports that no interference or instrumental bias significantly affects the accuracy of our measurements. The average 2SD for these δ^44/42^Ca and δ^43/42^Ca values is represented by the blue cross in the lower right of the graph (0.14‰ and 0.29‰, respectively). Data from this study are also reported in Tables S1 and S2.

### 3.2 LARS Reindeer

Reindeer tooth enamel and bone δ^44/42^Ca values range from −1.33 ± 0.07 (2SE, *n* = 3) to −1.02 ± 0.04 (2SE, *n* = 3) (Figure 2 and Table S1). For three specimens, M1 and M3 δ^44/42^Ca values are similar, while for the other two specimens, M1 δ^44/42^Ca values are more negative than M3 values (0.14‰ and 0.11‰ lower for E22‐083B and N22‐144, respectively). E22‐083A, the 10‐year‐old female with two nursing periods (2016 and 2019) has bone δ^44/42^Ca values 0.21‰ below M3 values. However, E22‐083B, the 7‐year‐old female with three nursing periods (2018, 2019, and 2020), shows no significant difference between bone and M3 δ^44/42^Ca values (the bone value is 0.08‰ higher than the M3 but within measurement uncertainty). The δ^44/42^Ca bone value of male specimens E22‐131, N22‐133, and N22‐144 (6+, 4+, and 5+ years old, respectively) is very similar to their M3 value with differences of −0.10‰, +0.10‰, and −0.004‰, respectively (Figure 2). All specimens have identical absolute bone δ^44/42^Ca values within a narrow range of ±0.01‰, except E22‐083A which has a bone value lower of 0.17‰.

**FIGURE 2:**
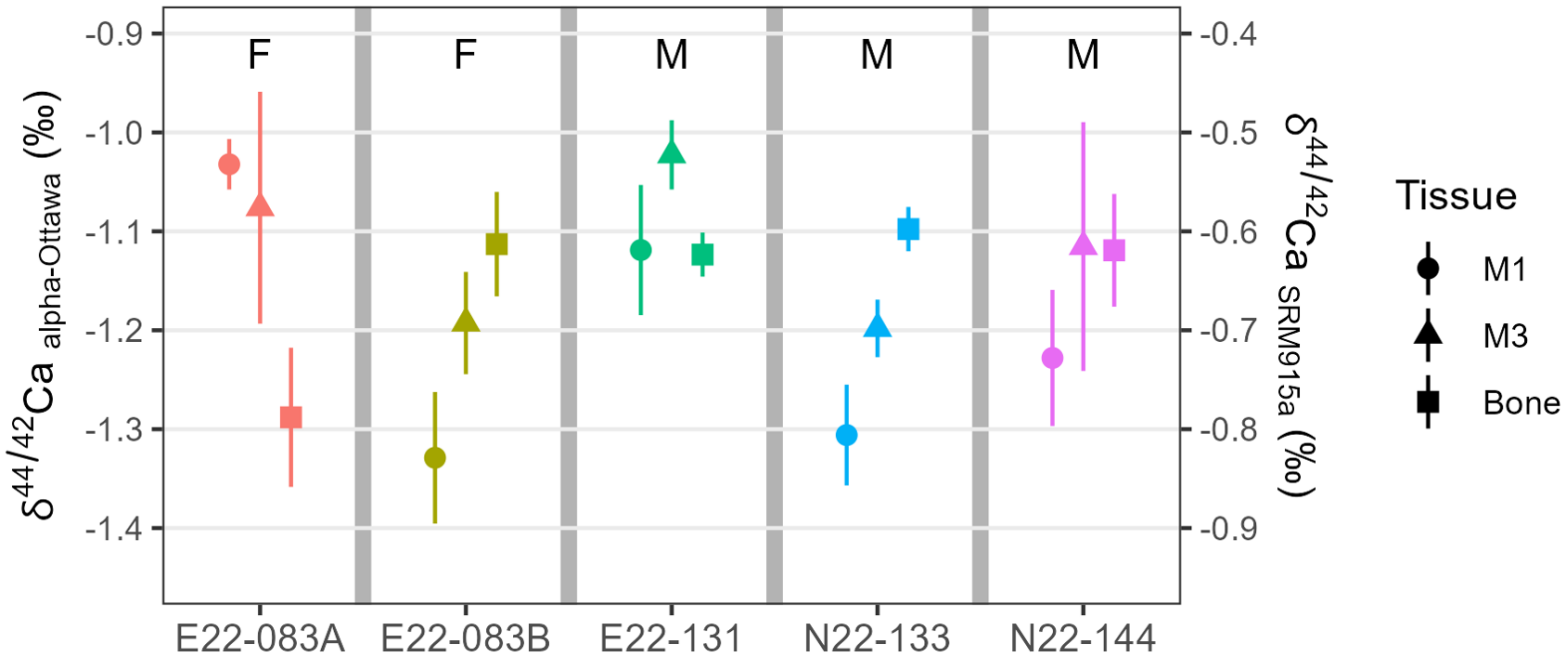
Reindeer δ^44/42^Ca values organized by specimen and tissues. δ^44/42^Ca values are expressed relative to our in‐house standard alpha‐Ottawa scale (left) and the SRM915a scale (right). For each specimen, tissues are organized from the earliest mineralization timeframe (M1, juvenile) to the latest (bone, wide adult timeframe), with M3 values as intermediate postweaning and pre‐reproduction adult references (corresponding to about 1 to 1.5 year post birth). The ID of each specimen is indicated on the *x*‐axis, and their respective sex is noted in top part of the graph (F = Female, M = Male). Error bars are given as ±2 standard errors of the mean (2SE). These data are also reported in the supplementary Table S1.

## 4 Discussion

### 4.1 Sampling Strategy and Lactation Effect

Contrary to our initial hypothesis, nursing does not appear to create significant offset between the δ^44/42^Ca values of the jawbones and M3 of female reindeer. Specimen E22‐083A even showed an inverse trend relative to the expected relationship with a 0.21‰ decrease despite two recorded nursing periods in the 6 years prior to death (Figure 2). Conversely, E22‐083B displayed only a negligible +0.08‰ difference (with overlapping confidence intervals) between bone and M3 and an absolute jawbone δ^44/42^Ca value falling within the observed range of non‐nursing male controls (Figure 2). Again, this is surprising considering the recent nursing activity of this specimen, with three consecutive nursing periods between 2 and 5 years before death (Table 1). Beyond M3 to bone comparisons, females do not have particularly ^44^Ca enriched bones (i.e., +δ^44/42^Ca values) compared to males (Figure 2), which contrast with observations in sheep, red deer, and mice [43, 44, 47]. Therefore, comparisons between M3 and jawbone δ^44/42^Ca values of a female individual does not provide clear insights into nursing and fertility for reindeer.

To explain this observation, it should be questioned if the small specimen count (*n* = 5, with only two females) has hindered the statistical power of this study. Previous studies have reported significant isotopic overlap between sexes and high individual variability, even when an average nursing enrichment was significant at the scale of a population, which is attributed to signal damping in bones and interspecimen nursing, diet, and physiological variability [43, 44, 47]. However, the more longitudinal M3‐to‐bone approach deployed in this work was designed to go beyond these population‐wide trends in bone values by quantifying individual baselines and nursing enrichments for each specimen (see Section 2). In this context, the absence of significant nursing signals cannot be satisfactorily explained exclusively through the lens of the small specimen count included here.

We instead propose three non‐mutually exclusive hypotheses to explain this observation. First, we identify potential shortcomings in the single‐point sampling strategy of M3 teeth. The similarity of diet offered to all studied specimens during their lifetime is likely the origin of the narrow range of bone δ^44/42^Ca values observed across most specimens (E22‐083A excluded, Figure 2). It supports that the average dietary Ca isotopic composition was consistent across specimens within the temporal window captured by jawbone's continuous remineralization, despite slightly distinct times of death (Table 1). However, despite this consistent average diet across specimens and resulting bone values, we observe fluctuating M3‐to‐bone isotopic offsets among control male specimens. These are mainly driven by the interspecimen variability of M3 values, at least in the case of control males (Figure 2). This suggests that limiting M3 sampling to a single small surface point has revealed non‐negligible transient changes in diet δ^44/42^Ca values (similar to [44]), which is suboptimal for establishing the stable postweaning and pre‐reproduction adult M3 baseline necessary to evaluate M3‐to‐bone nursing effects. The standardization of the M3 sampling area for all specimens was meant to limit the variability of M3 values (see Section 2). However, because enamel mineralization timings can slightly fluctuate between individuals [54, 58] and because all specimens are born a different year (Table 1), each M3 sample from this study is representative of a different point in time by constituting a snapshot of when this enamel mineralized [22, 44, 54, 58, 82]. These snapshots can reveal transient changes in diet source proportion or isotopic compositions [44], for example, seasonally and even with an isotopically similar average diet between specimens, which limits the reliability of such baseline for quantifying M3‐to‐bone ^44^Ca nursing enrichment. Therefore, future investigations into nursing signals should either prioritize intra‐M3 serial sampling to visualize the range of isotopic variations during the period of tooth formation or involve a larger single‐point M3 sampling strategy (i.e., incorporating the full crown height or a standardized portion of it) to represent a more time‐averaged sample. The latter approach would mitigate month‐to‐month deviations from the specimen's annual average. Sampling longitudinally along the tooth growth axis and ensuring consistency in length and distance to the cementoenamel junction across specimens would be the most appropriate single‐point sampling strategy. Important interyear variations of dietary δ^44/42^Ca values could still challenge the reliability of specimen‐specific M3 baselines for comparison with bone values, but differences between M3 and bone values in lactation‐free males would allow to identify such contexts. Nevertheless, the noisy M3 “baseline” described here is not a sufficient explanation of why specimen E22‐083B does not display a more significant nursing effect. Indeed, E22‐083B displays among the lowest M3 δ^44/42^Ca values of the studied population (Figure 2). This suggests a possible undershooting of its M3 baseline compared to its year average which would inflate rather than underestimate a M3‐to‐bone ^44^Ca nursing enrichment. However, this specimen still displays a nonsignificant M3‐to‐bone ^44^Ca nursing enrichment (i.e., within measurement uncertainty) despite its consequent nursing history (Table 1). This supports alternative hypotheses.

A second hypothesis to consider is whether reindeer nursing behavior and physiology can be expected to produce significant ^44^Ca enrichment in female blood and bones. ^44^Ca enrichment in female blood and bones are species and individual‐specific, as it varies with the amount of milk produced per nursing period, its Ca concentration and isotopic composition (i.e., blood to milk isotopic fractionation), the relative length of nursing periods, and their cumulative incidence [43, 45, 46, 47, 48]. It may be that these specific aspects of reindeer life history are not such that they can produce these offsets consistently. However, red deer ( *Cervus elaphus* ), a species phylogenetically close from reindeers with comparable lifespan and nursing duration [63, 64, 65, 83, 84], exhibit consistent milk Ca isotope fractionation (Δ^44/42^Ca_milk‐diet_ ≈ −0.6‰ [46]) and ^44^Ca enrichments in bones of females suspected of recent nursing activity [44]. This second hypothesis is thus unlikely to fully explain the lack of M3 to bone ^44^Ca enrichment in studied female reindeers, although individual differences in experienced nursing incidence, length, and proximity to death (only controlled indirectly in Hassler et al., 2021a [44]), or differences of Ca dietary availability, could have contributed to this divergence of results. The possession of antlers by female reindeer (a specificity among cervids) and its putative effects on Ca fluxes could also be a possible source of divergence. However, it must be noted that preliminary results on male red deer suggest limited effects of antlerogenesis on jawbone Ca isotopic composition for non‐lactating animals [44]. Ultimately, longitudinally sampling blood, milk, and antlers of reindeer during nursing while monitoring their Ca fluxes (e.g., similar to Hassler et al., 2021b [45] for pigs) is one of the only approaches that could allow to fully untangle the different underlying possibilities of this hypothesis.

Finally, our third hypothesis is that jawbones rapid remodeling might erase ^44^Ca nursing enrichment post nursing after a relatively short time. Bone turnover rate is very poorly constrained in the literature, with most studies on large mammals assuming an average elemental turnover rates in cortical bones of years to decades by lack of better species, age, bone, or specimen‐specific estimations (e.g., [70, 73, 74, 75, 76, 77, 85, 86, 87, 88]). To date, no study has quantified the spatial and temporal variations of Ca turnover in jawbones of reindeer or to our knowledge of any large mammals. It is likely that jawbone mineral turnover rate is faster than estimated prior to this study, potentially preventing the preservation of ^44^Ca enrichments beyond a few months or year post nursing. For example, specimen E22‐083B shows no significant ^44^Ca jawbone enrichment after spending only 2 years since its last nursing period. This hypothesis was also proposed in a previous study on red deer [44], where only females suspected of very recent nursing (less than 1 year) exhibited significant ^44^Ca enrichment in jawbones, whereas one female specimen suspected of more distant nursing (more than 1 year) showed jawbone δ^44/42^Ca values overlapping with males and nulliparous females. Furthermore, jawbone remodeling in female reindeer may be accelerated by annual antler growth cycles (a specificity of reindeer among female cervids), because antler growth stimulates bone resorption and/or accretion in some bones [87, 88]. More indirectly, human and canine studies indicate that jawbones may remodel more actively than the bulk average of cortical bones [89, 90], albeit with contrasting observations at low spatial resolution [91]. Together, these data suggest that jawbone Ca turnover in reindeer may be too rapid to retain long‐term nursing records beyond 1 year. More generally, this also highlights the need for better characterization of bone and tissue elemental turnover rates in parallel from developing the use of isotopic proxies. In this endeavor, methods of elemental kinetics using stable or radioactive tracers [92, 93], or novel methods of high‐resolution imaging techniques, such as micro‐CT and HR‐pQCT [94], could provide promising non‐invasive in vivo perspectives. In the absence of a better characterization of bone turnover rates, studies seeking to identify isotopic nursing signal could be proposed for other mammal species by instead targeting continuously growing tissues with well‐anchored chronology for high‐resolution sampling (e.g., ever‐growing tooth enamel or dentin; e.g., Martin et al., 2023 [95]).

### 4.2 Pathological Insights in E22‐083A

Apart from specimen E22‐083A, all specimens exhibit bone δ^44/42^Ca values confined to a narrow range of ±0.01‰. This is consistent with specimens sharing the same living environment with access to the same food sources regardless of age or sex and a potentially only time‐limited influence of nursing in females (see previous section). However, the bone δ^44/42^Ca value of individual E22‐083A contrasts with the rest of the population, being 0.21‰ lower than its own M3 value and 0.17‰ lower than the average bone isotopic composition of the other specimens (Figure 2). If this specimen had preserved the majority of jawbone minerals dating from between its birth and weaning age (e.g., delayed weaning or early death scenarios), this could, in theory, result in similar isotopic observations [44, 96]. However, this specimen lived to 10 years old and is the oldest individual in our dataset (Table 1). Additionally, the M1 and M3 δ^44/42^Ca values of this specimen are positioned in the higher bracket of the population, showing a clear postweaning signal (Figure 2). This clearly contradicts the above‐mentioned theory and suggests alternative causes for the low bone δ^44/42^Ca value of E22‐083A.

Certain pathological conditions, such as bone diseases (e.g., osteoporosis) or disruptions in renal Ca fluxes, have been shown to lower δ^44/42^Ca values in blood and urine [37, 42, 97, 98, 99, 100, 101, 102, 103, 104], an isotopic trend plausibly conveyed to bones through remodeling. No report of illness suggesting weakened bones (i.e., osteoporosis) or renal disfunctions was reported for E22‐083A by LARS technicians, nor clear evidence of disease was observed on the jawbone itself. However, the specimen's advanced age increased the likelihood of such pathologies to have occurred, in a context where some of these pathologies (e.g., osteoporosis) are known to progress silently until advanced stages, notably for humans [105]. To our knowledge, no other physiological or environmental process can plausibly explain this observed bone value anomaly. Thus, these pathologies remain our most parsimonious hypothesis, despite the lack of direct clinical evidence. This result calls for more investigations into how pathologies and aging affects bone δ^44/42^Ca values. It also suggests some degree of caution for building ecological interpretations based on bone δ^44/42^Ca values from biologically old *Rangifer* specimens (instead of e.g., M3 values [44]) and supports the complementary use of ageing methods (e.g., dental aging [58]) for cases where bone δ^44/42^Ca values are investigated and ages at death are unknown.

### 4.3 Milk Consumption by Juveniles

For each specimen, the M1 δ^44/42^Ca value is either indistinguishable or more negative than the specimen's M3 and bone δ^44/42^Ca values (Figure 2), with the sole exception of E22‐083A bone value disccused in the previous section. The formation of *Rangifer* M1 tooth enamel occurs in utero and during the initial months following birth, as supported by tooth eruption timings [54, 56, 58, 59], radiographic evidence in *Rangifer* and other cervids [55, 62], and geochemical clues from Pleistocene reindeer [44, 54]. This mineralization period coincides with juvenile Ca intakes characterized by lower δ^44/42^Ca values than corresponding adult diets, especially maternal milk and, to a lesser extent, placental blood transfers [42, 45, 46]. Therefore, early forming M1 enamel is expected to exhibit more negative δ^44/42^Ca values compared to later‐forming tissues (e.g., M3 enamel, bone remodeling during adulthood). The absence of these more negative M1 δ^44/42^Ca values in some specimens can appear unusual (Figure 2) but can be directly attributed to the higher degree of enamel wear of these specimens. The tip of the crown of a tooth is the first part of the enamel to mineralize but also the first to wear [62], progressively erasing the earliest mineralization records. This is exemplified in specimens E22‐083B and N22‐133, which display the most unambiguous negative M1 δ^44/42^Ca values relative to their M3 (Figure 2) while possessing the least worn M1 crowns among the studied specimens (Table 1). In these cases, the M1 tip (the sampling point) was located more than 0.7 cm above the cementoenamel junction (the enamel base). In contrast, all other specimens (E22‐083A, E22‐131 and N22‐144) retained less than 0.7 cm of enamel from base to tip (ranging from 0.51 to 0.67 cm, Table 1). This degree of dental wear was presumably enough to erase the pre‐weaning breastfeeding signals in the M1 enamel of these three specimens.

These findings indicate that M1 tip sampling is not a reliable method for establishing specimen‐specific pre‐weaning baselines in *Rangifer*. It also suggests that studying *Rangifer* weaning transitions using M1 spatial sampling would be possible only in specimens with well‐preserved M1 crowns (e.g., young specimens), delayed M1 formation, or later weaning ages than studied specimens. Nonetheless, these results align with previous reports of early‐mineralizing enamel in humans [96, 106, 107], sheep [108], red deer, and Pleistocene reindeer [44], which display pre‐weaning low δ^44/42^Ca values when enamel wear level and tooth mineralization timing allow.

## 5 Conclusion

This study on reindeer raised in a controlled environment reports data consistent with previous observations (from cervids and other vertebrates) with Ca isotopes varying with diet (including milk consumption during nursing) and possibly some pathologies. However, the study failed to identify a persistent nursing‐driven ^44^Ca enrichment in female specimens. Consequently, the proposed method (i.e., M1, M3 and jawbone single‐point samplings for δ^44/42^Ca intraspecimen comparisons) is not a reliable and scalable framework to estimate past and present reindeer nursing history. We formulated three hypotheses that can explain, to different degrees, the absence of observable nursing‐driven ^44^Ca enrichment in the studied reindeer population: (1) a suboptimal M3 sampling strategy, (2) possible differences in experienced nursing activity or associated isotopic fractionation of studied reindeer compared to other mammal populations from the literature, and (3) a likely fast bone recycling in reindeer jawbone, achieving a Ca turnover rate rapid enough to erase any significant Ca isotope nursing signal within less than 2 years postnursing. We suggest that a more robust understanding of Ca turnover rates in reindeer skeletal elements would allow the design of better sampling strategies for future studies, to target bones or bone regions with the highest potential for preserving nursing‐driven ^44^Ca enrichments. More generally, this study highlights the need to quantify elemental turnover rates in skeletal elements of target species for isotope ecology and paleoecology applications.

## Author Contributions

**Auguste Hassler:** conceptualization, investigation, funding acquisition, writing – original draft, methodology, writing – review and editing, visualization, validation. **Eve E. Lindroos:** investigation, writing – review and editing. **Lu Yang:** methodology, writing – review and editing. **Zoltan Mester:** writing – review and editing. **Kate Britton:** writing – review and editing, conceptualization, supervision, funding acquisition. **Clément P. Bataille:** writing – review and editing, conceptualization, supervision, funding acquisition.

## Conflicts of Interest

The authors declare no conflicts of interest.

## Supporting information




**Table S1:** Reindeer mean, double standard deviation (SD), and standard error (SE) of δ^44/42^Ca and δ^43/42^Ca values (relative to alpha‐Ottawa; this study). Mean δ^44/42^Ca values are also reported relative to the SRM915a scale, following the conversion formula established by Hassler et al. (2026) [78].
**Table S2:** Reference material mean, double standard deviation (SD), and standard error (SE) of δ^44/42^Ca and δ^43/42^Ca values (relative to alpha‐Ottawa; this study; subset of Hassler et al., 2026 [78]). Mean δ^44/42^Ca values are also reported relative to the SRM915a scale, following the conversion formula established by Hassler et al. (2026) [78].

## Data Availability

The data supporting this article have been included as part of the Supporting Information.
